# Prevalence and Predictors of Musculoskeletal Pain in Recreational Resistance Trainers: Associations with Age, Gender, and Training History

**DOI:** 10.3390/sports14030087

**Published:** 2026-02-26

**Authors:** Dimitar Shabanliyski, Denise Soares, Karim Abbady, Suat Kasap

**Affiliations:** 1 Liberal Arts Department, American University of the Middle East, Egaila 54200, Kuwait; dimitar.shabanliyski@aum.edu.kw; 2 College of Engineering and Technology, American University of the Middle East, Egaila 54200, Kuwait; kareem.abbady@aum.edu.kw (K.A.); suat.kasap@aum.edu.kw (S.K.)

**Keywords:** musculoskeletal pain, resistance training, recreational weightlifting, low back pain, shoulder pain, exercise-related injuries, physical activity, injury prevalence, adult population

## Abstract

Recreational weightlifting has become increasingly popular in Kuwait, yet limited data exist regarding musculoskeletal pain (MSP) associated with it. This study aimed to assess the prevalence and anatomical distribution of MSP among recreational resistance trainers (RRTs) in Kuwait and to examine pain patterns according to gender, age, and training history. A total of 642 RRTs (304 males and 341 females) who practiced resistance training for at least 6 months 2 times a week were surveyed using the Nordic Musculoskeletal Questionnaire (NMQ) through face-to-face interviews. MSP prevalence over the previous 12 months was analyzed by anatomical region, and binary logistic regression was applied to investigate predictors of low back pain (LBP). The results indicated a high prevalence of MSP, particularly in the lower back and shoulders, with increased reports among older participants and those with longer training histories. Logistic regression analysis revealed that age and years of practice were significant predictors of LBP, while female gender was associated with higher odds of reporting symptoms. These findings highlight the substantial burden of MSP among RRTs in Kuwait and underscore the importance of targeted preventive strategies, including training supervision, technique optimization, and age-specific interventions, to reduce injury risk and promote long-term musculoskeletal health.

## 1. Introduction

The term “musculoskeletal pain” (MSP) refers to a group of conditions that affect the muscles, tendons, ligaments, joints, blood vessels, cartilage, nerves, spinal discs, or tissues, including those associated with abnormalities that can be caused or exacerbated by physical activity [1]. Globally, MSP is a significant public health issue that causes a great deal of suffering and impairment [2]. Most sports-related injuries are musculoskeletal in nature. More than half of all sports-related injuries in adolescents and young adults are caused by overuse of the musculoskeletal system [3]. All individuals, regardless of age, gender, or socioeconomic background, have had one or more bouts of MSP or injury at some point in their lives. Approximately 47% of people are generally affected, and from these, between 39% and 45% have persistent issues that require medical attention. An MSP that is not correctly controlled can have a severe negative impact on quality of life as well as serious socioeconomic issues [4].

Activities involving repetitive movements, heavy lifting, awkward postures, and excessive mechanical load are well-established risk factors for MSP, particularly affecting the lower back, neck, shoulders, and upper extremities. Despite its benefits, recreational weightlifting is associated with a considerable risk of MSP, with the lower back, shoulder, and knee joints being the most frequently affected regions [5,6]. Notably, shoulder injuries alone account for approximately 36% of all injuries, and MSP was reported among resistance training participants [7], underscoring the need for systematic monitoring and prevention strategies.

Recreational resistance trainers (RRTs) are defined as individuals engaging in structured resistance exercise at least twice per week for general fitness purposes without competitive intent [8]. RRTs constitute a population at increased risk due to repeated exposure to high loads, repetitive training patterns, and suboptimal movement mechanics [8]. Monitoring musculoskeletal symptoms in such populations is therefore essential for identifying high-risk body regions and informing preventive strategies.

One of the most widely used tools for assessing musculoskeletal symptoms is the Nordic Musculoskeletal Questionnaire (NMQ), a standardized instrument developed for epidemiological research [9]. The NMQ enables systematic evaluation of MSP and discomfort across nine body regions and captures symptoms experienced over specified time frames, such as the previous 12 months and the past 7 days. By identifying the prevalence and anatomical distribution of MSP, the NMQ provides valuable information for understanding pain patterns and supporting targeted interventions to reduce the burden of MSPs [10]. The NMQ has been translated into multiple languages, including Arabic, and validated in various countries around the world to ensure its applicability and reliability in different cultural contexts and occupational environments [11].

Kuwait is a member of the Gulf Cooperation Council (GCC), characterized by consistently high ambient temperatures that pose a significant challenge to regular outdoor physical activity. Despite the public health relevance of this issue, there are few studies examining the daily habits of the Kuwaiti population, particularly with respect to physical activity levels. Available evidence indicates that only 56% of males and 24% of females meet the recommended physical activity guidelines, highlighting a substantial prevalence of physical inactivity within the population [12].

Excellent economic opportunities, notably in the fitness sector, are enabled by Kuwait’s wealth and rapid growth in oil revenues. The opening of new fitness facilities is one of the economic opportunities enabled by the government’s liberalization and privatization initiatives. The prospective expansion of the fitness business is supported by this economic environment [13]. Kuwait City’s rapid urbanization and population growth have changed people’s lifestyles and raised demand for recreational amenities like gyms as well. High-density development has historically been encouraged by urban planning decisions, which may increase the demand for exercise facilities to handle the expanding population [14]. Another factor is that more people are participating in fitness activities because of the general public’s growing understanding of the advantages of physical activity in recent years. Gyms are crucial to the community since they are recognized as the main indoor spaces for engaging in physical activity [15].

While the effects of age, gender, and training exposure on musculoskeletal conditions such as low back pain (LBP) have been explored in general populations, there are limited data describing these associations within physically active or resistance training populations, particularly in the GCC [12]. Most existing studies focus on heterogeneous adult samples or clinical cohorts, rather than on individuals who engage in regular structured resistance training (e.g., ≥2 sessions/week) [4,12]. Evidence from fitness and sports sciences suggests that physically active populations may exhibit distinct patterns of pain prevalence and risk factors compared to less active or clinical groups, and that resistance training itself can influence musculoskeletal health [5,16,17]. By focusing on RRTs—a group underrepresented in the regional literature—this study provides novel insight into how demographic and training characteristics relate to MSP in an active, non-clinical GCC population. Considering this, this study aimed to assess the prevalence and predictors of musculoskeletal pain in recreational resistance trainers, examining how age, gender, and training history influence the occurrence of pain in this population.

## 2. Materials and Methods

### 2.1. Sample Description

The sample included 645 recreational resistance trainers (RRTs) regularly attending fitness centres in Kuwait. Eligible participants had engaged in gym-based resistance training at least twice per week for a minimum of six months. A total of 642 participants met the inclusion criteria and completed the survey: 304 males (mean age = 37.50 ± 7.80 years) and 341 females (mean age = 34.45 ± 7.55 years). The average height of the interviewed males is 178.28 cm (±6.70 cm) and the average weight is 79.30 (±16.10 kg). The average height of the females is 162.8 (±6.40 cm) and the average weight is 62.5 (±11.10 kg) (Table 1).

### 2.2. Procedures

Data were collected from RRTs residing in the Kuwaiti metropolitan area who were selected via convenience sampling from various gyms and health clubs (a total of 15 locations) using the pretested NMQ via face-to-face interviews from December 2023 to April 2025. This study was conducted in accordance with the ethical standards of the American University of the Middle East (AUM). The research protocol was reviewed and approved by the AUM Research Department, under approval code F-RFD-08/Nov2023. All participants provided informed consent after receiving a detailed explanation of the study’s objectives, procedures, and data confidentiality measures.

### 2.3. Data Analysis

The RRTs observed have been divided into categories according to their gender, age and years of practice. For age, the distribution is in 4 categories—AGE A for practitioners under 25 years old (85 participants), AGE B 25–34 years old (285 participants), AGE C from 35 to 45 years old (202 participants) and AGE D more than 45 years old (69 participants). In relation to the years of continuous practicing, the following categories have been created—History A for exercising less than a year (208 participants), History B 1–5 years (193 participants) and History C for practicing more than 5 years (241 participants). Age and years of resistance training practice were categorized into ordinal groups to account for potential nonlinear associations with LBP and to enhance interpretability across meaningful life stages and experience levels, consistent with common epidemiological practice.

To examine the association between the occurrence of LBP and three categorical predictors—age, gender, and history of resistance training practice—a binary logistic regression analysis was performed. The dependent variable was the presence or absence of self-reported LBP. All predictors were treated as categorical variables and entered using dummy coding. For age, participants were divided into four predefined age groups, with the youngest group (Age Group A) set as the reference category. Gender was coded as male or female, with female used as the reference. The history of practice was categorized into three groups based on the duration of regular resistance training, with the group representing the shortest training history (Group A) serving as the reference. This coding allowed for the comparison of odds of experiencing LBP in each subgroup relative to the corresponding reference category. As the analysis used binary logistic regression with categorical predictors and a binary outcome, assessment of normality was not required, since logistic regression does not assume normally distributed variables.

Results were presented as regression coefficients (B), Wald χ^2^ statistics, associated *p*-values, and odds ratios [Exp(B)] with 95% confidence intervals. A *p*-value < 0.05 was considered statistically significant. Model fit was assessed using the Omnibus Test of Model Coefficients, and explanatory power was evaluated with the Nagelkerke R^2^. Classification accuracy was also reported. To assess multicollinearity, a linear regression with the same predictors was performed and variance inflation factors (VIFs) were examined. Model calibration was verified using the Hosmer–Lemeshow goodness-of-fit test. Influential observations were screened by inspecting standardized residuals and Cook’s distance, and missing data were handled using listwise deletion. All analyses were conducted using IBM SPSS Statistics version 29.

## 3. Results

**Figure 1:**
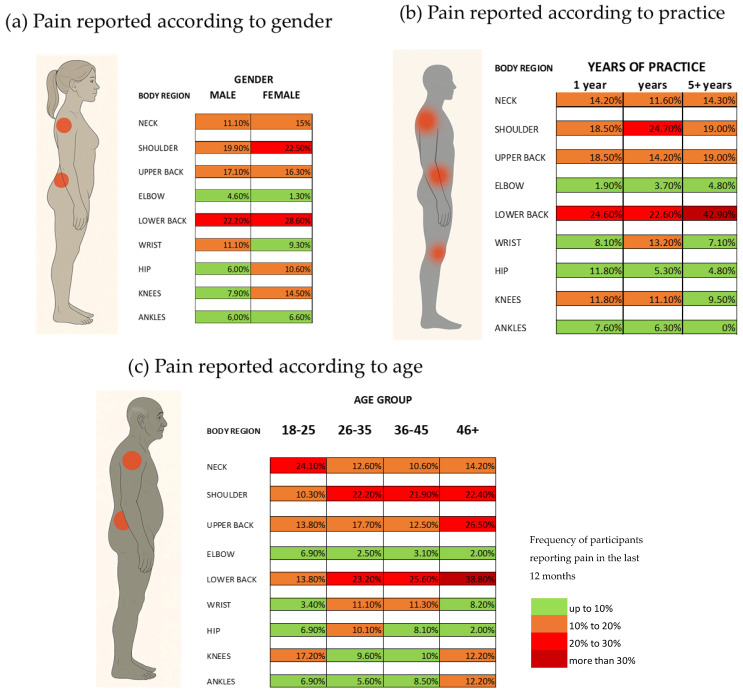
Prevalence of MSP reported in the past 12 months, by anatomical region. (**a**) stratified by gender, (**b**) by years of resistance training practice, and (**c**) by age group.

Figure 1 presents the results from the prevalence of MSP symptoms in the anatomical region in the last year according to gender, years of practice, and age group. The results were grouped into frequency of participants referring to pain at that anatomical region (less than 10%, 10–20%, 20–30% and more than 30% of the participants).

When analyzing gender differences, 20–30% of participants referred to LBP, independently of gender. A total of 22.50% of female subjects also referred to pain in the shoulders in the last 12 months (Figure 1a). For the years of continuous practice of regular exercise, 20–30% of participants with less than 1 year and 1–5 years of practice referred to LBP. These numbers increase to almost 43% of practitioners with more than 5 years of practice, who also referred to LBP (Figure 1b). Participants with 1–5 years of practice also referred to pain in the shoulder region. The age groups analysis shows that LBP and shoulder pain are common in more than 23% of the participants aged 25 years and above. Participants between 18 and 24 referred to pain in the neck region (Figure 1c).

Table 2 shows the results for the logistic regression analysis in relation to the report of LBP in relation to gender, age, and time of practice. The model was statistically significant (χ^2^(4) = 20.35, *p* < 0.001), indicating that the predictors significantly explain variation in LBP occurrence. Also, the model explained 28% (Nagelkerke R^2^ = 0.28) of the variance in LBP occurrence and correctly classified 78% of cases, with a sensitivity of 80% and a specificity of 75%. The constant in the logistic regression model shows the probability of an individual in the reference category (18–24 years old, female, and less than 1 year of practice) showing a 14% of chance of experiencing LBP in the next 12 months.

Age: Age Group C (with Exp(B) = 4.113, sig 0.025) has significantly higher odds of experiencing LBP compared to the reference age group (18–24 years old), while the other age groups do not show significant results. Age Group C (35–45 years old) has a significant impact on LBP, with odds 4.11 times higher than the reference age group.

Gender: Being male significantly decreases the odds of LBP by 42.3% compared to females (Exp(B) = 0.577, sig 0.024).

History of Practice: History Group 2 (25–35 years old) has significantly higher odds (2.35 times) of experiencing LBP compared to the reference group, while History Group 1 has no significant effect.

## 4. Discussion

The purpose of this study was to analyze the prevalence and incidence of MSP symptoms in the recreational weightlifter population in Kuwait. Our results pointed out many symptoms related to MSP in most of the body joints of the subjects. The gender, age, and years of practice can affect the symptoms significantly.

The comparison between males and females shows that women reported more pain in the shoulders and lower back than males (Figure 1a). Previous research has shown that women may experience different pain responses to exercise. For instance, while both men and women show signs of muscle fatigue after repetitive upper limb tasks, the changes in pain sensitivity do not significantly differ between sexes [18]. This could have a hormonal influence, since female sex hormones play a significant role in the etiology of musculoskeletal degenerative diseases [19]. Hormonal pathways and shoulder morphology contribute to the increased susceptibility of women to shoulder injuries. These biological differences can affect the stability and strength of the shoulder joint, making women more prone to pain and injury [20]. Postmenopausal women experience accelerated disc degeneration due to estrogen deficiency, leading to narrower intervertebral disc spaces, increased prevalence of spondylolisthesis, and facet joint osteoarthritis. This hormonal change is associated with a higher prevalence of LBP in postmenopausal women compared to age-matched men [21]. Women with LBP often report higher levels of stress and psychological distress compared to men. This psychological burden can exacerbate the perception and reporting of pain [22].

Analyzing the MSP symptoms according to years of practice, the results show that the incidence of LBP increases with the years of practice (Figure 1b). Increased physical activity can lead to muscle contraction and stress on structures such as muscles, ligaments, joints, and intervertebral discs, contributing to LBP [23]. This is particularly relevant in activities that involve repetitive movements or prolonged periods of exertion [24]. Engaging in sports or activities that involve repetitive movements can lead to overuse injuries. These repetitive actions place continuous strain on the spine and surrounding muscular and ligamentous structures, increasing the risk of LBP [24]. Higher training intensity and greater time under tension during exercise are associated with lower pain intensity over time, suggesting that, while these factors can initially contribute to LBP, they may also play a role in its management [25,26].

Another interesting result is that most people reported shoulder pain, independent of years of practice (Figure 1b). Shoulder pain can result from biomechanical dysfunction and the inherent instability of the shoulder joint, which leads to functional overload of various tissues [27]. This complexity means that pain can arise regardless of the duration of practice. Proper technique during activities such as resistance training is crucial to avoid shoulder pain. Poor technique, rather than the amount of time spent practicing, is a significant risk factor for injury [28].

Analyzing the reported pain according to age, younger people reported more pain in the neck region than older subjects (Figure 1c). Younger individuals may experience impaired postural control and proprioception, which can lead to increased neck pain during physical activities. This is supported by findings that neck pain can impair postural stability and increase postural sway, particularly in dynamic activities [29].

Our results showed that the prevalence of people reporting LBP increases to almost 40% in subjects more than 45 years old. This is in accordance with previous studies showing that the prevalence of LBP increases with age, and it is particularly high among those aged 45 and older [30,31]. This age group often experiences chronic LBP, which is a significant cause of disability. Older adults often have comorbid conditions that can complicate the management of LBP. These conditions can make it difficult to engage in exercise without experiencing pain [32].

In the logistic regression analysis for LBP, age and training history emerged as significant predictors of symptom occurrence. Specifically, individuals aged 35–45 years (Age Group C) were 4.11 times more likely to report LBP compared to those aged 18–27, while those with over 5 years of recreational weightlifting experience had 2.35 times the odds of reporting symptoms compared to beginners. Gender also showed a protective association, with males being 42% less likely to report LBP. Studies have reported significantly increased odds of LBP in individuals older than 40 years compared to younger adults, likely reflecting cumulative tissue loading over time. Likewise, the lower odds of LBP in males aligns with broader population research indicating that females have higher LBP prevalence than males, potentially due to anatomical, hormonal, and pain processing differences [33].

From a clinical standpoint, these findings suggest that the risk of LBP increases non-linearly with age, particularly entering middle adulthood, a stage commonly associated with accumulated musculoskeletal strain [34,35]. The elevated odds in the group with >5 years of training may reflect chronic exposure to biomechanical stressors, emphasizing the need for technique supervision and periodic training load assessment, even in recreational populations. The gender difference, while possibly influenced by reporting bias or physiological differences, highlights the importance of tailoring prevention strategies—particularly for female gym users.

As an illustrative model-based estimate, an individual who is female, 35–45 years old, and has been consistently weight training for 1–5 years would fall into a moderate-risk profile (estimated probability ≈ 47.9%). While male gender lowers the risk, middle-aged status and accumulated training time increase vulnerability. As the logistic regression reflects associations within a cross-sectional sample and includes a limited set of predictors, these probabilities indicate relative likelihoods within the study population, not precise clinical risk for a specific individual. When applied carefully, these results are clinically meaningful for gym professionals, physiotherapists, and public health planners aiming to prevent LBP through age- and experience-specific strategies, including mobility screenings, load adjustments, and core stabilization programmes.

This study presents several limitations that must be acknowledged. First, the type of workout—specifically the duration, intensity, supervision, and exact exercise modalities—was not controlled or recorded. While inclusion criteria required participants to engage in recreational weightlifting activities at least twice per week, which contributed to a degree of sample homogeneity, the lack of detailed training variables may affect the precision of associations with musculoskeletal symptoms. Second, the NMQ used in this study captures only the presence or absence of symptoms, without accounting for pain intensity, duration, or functional impact, limiting interpretation of the clinical severity. Third, important individual health data—such as BMI, occupation, comorbidities, and injury history—were not collected, potentially contributing to residual confounding in the logistic regression models. Finally, nationality was not recorded. Given that approximately 70% of Kuwait’s population consists of expatriates, the findings cannot be generalized to Kuwaiti nationals alone. Instead, they reflect the broader population of individuals living in Kuwait and regularly attending gyms. Future research should address these gaps to strengthen the external and internal validity of findings on RRTs.

## 5. Conclusions

This study investigated the prevalence of musculoskeletal symptoms (MSS) among recreational resistance trainers (RRTs) in Kuwait and identified demographic and training-related factors associated with these symptoms. Musculoskeletal pain was commonly reported, particularly in the lower back, shoulders, and knees, reflecting areas of the body most stressed during resistance training.

Focusing on low back pain (LBP), logistic regression analysis revealed that age and training history were significant predictors of LBP. Specifically, individuals aged 35–45 and those with more than 5 years of training experience had higher odds of reporting LBP. Conversely, male participants exhibited lower odds of experiencing LBP compared to females. These findings highlight that, even within an active, non-clinical population, individual characteristics influence the likelihood of developing LBP. Future studies should incorporate training volume, exercise technique, and supervision variables to better understand modifiable risk factors in this population.

## Figures and Tables

**Table 1:** Mean and Standard deviation (Mean ± SD) of the sample description variables.

Sample	Age (Years) (Mean ± SD)	Height (cm) (Mean ± SD)	Weight (kg) (Mean ± SD)
Male (*n* = 304)	37.50 (7.80)	178.28 (6.70)	79.3 (16.10)
Female (*n* = 341)	34.45 (7.55)	162.80 (6.40)	62.5 (11.10)

Notes: *n*, number of participants.

**Table 2:** Logistic regression analysispredictors of LBP according to age group, gender and years of practice.

Predictor	B	S.E	Wald	Sig.	Exp(B) (Odds Ratio)	95% CI for Exp (B)
Age (ref = Age Group A) (*n* = 85)						
Age (B) (*n* = 285)	0.811	0.572	2.011	0.156	2.251	0.732–6.920
Age(C) (*n* = 202)	0.934	0.58	2.598	0.107	2.545	0.827–7.824
Age (D) (*n* = 69)	1.414	0.632	5.001	0.025 *	4.113	1.193–14.177
Gender (ref = Female) (*n* = 341)						
Male (*n* = 304)	−0.55	0.244	5.071	0.024 *	0.577	0.357–0.933
History of Practice (ref ≤ 1 year) (*n* = 208)						
History (1–5 years) (*n* = 193)	−0.01	0.249	0.002	0.967	0.99	0.609–1.610
History (>5 years) (*n* = 241)	0.856	0.389	4.851	0.028 *	2.354	1.098–5.046
Constant	−1.804	0.545	10	<0.001	0.165	

PS: AGE A: under 25 years old, AGE B: 25–34 years old, AGE C: from 35 to 45 years old, and AGE D: more than 45 years old. History A: for exercising less than a year, History B: 1–5 years, and History C: practicing more than 5 years. n: number of participants. * *p* < 0.05.

## Data Availability

Data are available upon request.

## Abbreviations

The following abbreviations are used in this manuscript:

NMQ	Nordic Musculoskeletal Questionnaire
MSP	Musculoskeletal Pain
LBP	Low Back Pain
RRTs	Recreational Resistance Trainers
